# Assessment of Medical Decision-Making Competence in Emergency Settings: Application of a Validated Scale in a Case Study

**DOI:** 10.1192/j.eurpsy.2025.1503

**Published:** 2025-08-26

**Authors:** V. Del Rio Peña, A. Pelegrí, M. Boadas i Gironés, A. Barnés Andreu, D. Vegas Renom, C. Fernández Alcobet, M. Aparicio Muñoz, C. Tablero Nadal, Á. Ferrer Alberti, F. Estrada Coma, E. Aguilar Morales, V. Soria Tomás, D. Palao Vidal

**Affiliations:** 1 Consorci Corporació Sanitària Parc Taulí de Sabadell, Sabadell (Barcelona), Spain

## Abstract

**Introduction:**

The assessment of competence for medical decision-making is a critical aspect in the care of patients with mental health disorders. Since patient autonomy is a fundamental pillar of medical ethics, evaluating their capacity to make informed decisions ensures respect for their rights while safeguarding their well-being. An accurate assessment of competence not only facilitates safer and more appropriate decisions for the patient, but also allows physicians to act ethically and legally. Understanding the tools and criteria for such assessments is essential to balance patient autonomy with the protection of their health.

**Objectives:**

Identify factors that may influence a patient’s competence for specific decisions.

Familiarize with validated scales for assessing patient competence.

Identify strategies to improve competence in cases where necessary.

**Methods:**

We present the case of a 65-year-old female patient without significant medical or psychiatric history, who was evaluated in the emergency department for cardiac tamponade, requiring urgent intervention via pericardiocentesis. At the time of the procedure, the patient refused the intervention due to severe pain, requesting voluntary discharge without undergoing further tests. Psychiatry was consulted to assess the patient’s decision-making capacity. An interview was conducted using the MacCAT-T (MacArthur Competence Assessment Tool for Treatment) scale.

**Results:**

A joint interview was conducted between the emergency medicine, intensive care, and psychiatry departments. The results indicated partial competence of the patient for this medical decision (understanding of the procedure and its impact on daily life, but high risk associated with the decision). The patient’s family was involved in the decision-making process, and it was decided to extend the emergency department stay for two additional days to promote better patient competence. No psychopathology was found that impaired the patient’s competence. Ultimately, it was determined that the patient had the necessary competence for this specific decision, and she was discharged home.

**Conclusions:**

Interviews assisted by validated competence assessment scales, such as the MacCAT-T, can be a useful tool in challenging decision-making contexts in emergency situations, providing a more objective and ethical evaluation of patient competence.

**Disclosure of Interest:**

None Declared

